# The role of the hospital bed in hospital-onset *Clostridioides difficile*: A retrospective study with mediation analysis

**DOI:** 10.1017/ice.2023.254

**Published:** 2024-05

**Authors:** Lucy S. Witt, Jessica Howard-Anderson, Radhika Prakash-Asrani, Elizabeth Overton, Jesse T. Jacob

**Affiliations:** 1 Division of Infectious Diseases, Department of Medicine, Emory University School of Medicine, Atlanta, Georgia; 2 Emory Healthcare, Atlanta, Georgia; 3 Emory Antibiotic Resistance Center, Atlanta, Georgia

## Abstract

**Objective:**

To determine whether residing in a hospital bed that previously held an occupant with *Clostridioides difficile* increases the risk of hospital-onset *C. difficile* infection (HO-CDI).

**Methods:**

In this retrospective cohort study, we used a real-time location system to track the movement of hospital beds in 2 academic hospitals from April 2018 to August 2019. We abstracted patient demographics, clinical characteristics, and *C. difficile* polymerase chain reaction (PCR) results from the medical record. We defined patients as being exposed to a potentially “contaminated” bed or room if, within the preceding 7 days from their HO-CDI diagnosis, they resided in a bed or room respectively, that held an occupant with *C. difficile* in the previous 90 days. We used multivariable logistic regression to determine whether residing in a contaminated bed was associated with HO-CDI after controlling for time at risk and requiring intensive care. We assessed mediation and interaction from a contaminated hospital room.

**Results:**

Of 25,032 hospital encounters with 18,860 unique patients, we identified 237 cases of HO-CDI. Exposure to a contaminated bed was associated with HO-CDI in unadjusted analyses (odds ratio [OR], 1.8; 95% confidence interval [CI], 1.4–2.31) and adjusted analyses (OR, 1.5; 95% CI, 1.2–2.0). Most of this effect was due to both mediation from and interaction with a contaminated hospital room.

**Conclusions:**

Residing in a hospital bed or room that previously had a patient with *C. difficile* increases the risk of HO-CDI. Increased attention to cleaning and disinfecting the healthcare environment may reduce hospital transmission of *C. difficile*.

*Clostridioides difficile* can cause a severe, often recurrent diarrheal illness, with an estimated 500,000 infections per year in the United States.^1^ This disease can be debilitating, and 1 in 6 patients will experience a recurrence within 2 months of their diagnosis. Preventing hospital-onset *C. difficile* infection (HO-CDI) remains a challenge because *C. difficile* spores can survive on surfaces for months, and intensive cleaning and disinfection are required to eradicate them.^2–6^ Many organizations, including the Agency for Healthcare Research and Quality and the US Centers for Disease Control and Prevention, have prioritized efforts to reduce healthcare transmission of *C. difficile*, and HO-CDI is a nationally reported marker of hospital quality.^2,7^

Healthcare exposures increase the risk of CDI. In prior research, *C. difficile* spores have been detected at multiple locations in hospital rooms, including the bed, floor, sink, and light switch, despite appropriate disinfection measures.^8–12^ These environmental reservoirs for *C. difficile* pose a risk to hospitalized patients placed in a room or hospital bed that previously held a patient with *C. difficile*. Prior studies have described an increased odds ratio of 1.1 to 4.5 for HO-CDI when residing in a room where a prior occupant was diagnosed with *C. difficile.* ^13,14^ However, to our knowledge, no studies have analyzed the risk of *C. difficile* transmission associated with being in a hospital bed that previously held someone with *C. difficile*. The US Food and Drug Administration received >700 reports of bed mattress covers failing to prevent blood or body fluids from leaking into mattresses between 2011 and 2016, making the bed a particularly concerning reservoir for *C. difficile*.^15^

In this retrospective, observational cohort study, we used novel, real-time bed trackers installed in 2 academic hospitals to determine whether residing in a hospital bed that previously held an occupant with *C. difficile* (ie, “contaminated bed”) increased the risk of HO-CDI. We then used mediation and interaction analyses to determine whether the relationship between a contaminated bed and HO-CDI was explained or modified by a patient being exposed to a contaminated hospital room (eg, a room where the prior occupant had *C. difficile*).

## Methods

### Healthcare setting

We used a real-time, radiofrequency- and infrared-light–based location system (AgileTrac, GE Healthcare, Wauwatosa, WI) installed on the subframe of hospital beds to track the movement of beds at 2 academic hospitals within the same healthcare system in Atlanta, Georgia. Hospital A is a 529-bed, academic–community, tertiary-care hospital with women’s health services. Hospital B is a 751-bed, academic, tertiary-care hospital that performs solid organ and hematopoietic stem-cell transplantation. All hospital rooms are single occupancy. At both study hospitals, the environmental hygiene protocols recommend daily cleaning and disinfecting of hospital rooms using a germicidal cleaner and more comprehensive terminal cleaning that involves wiping and disinfecting all surfaces. Rooms with a patient who tested positive for *C. difficile* were cleaned using either BruTab (Brulin, Indianapolis, IN; hospital A) or Oxycide (EcoLab, St. Paul, MN; hospital B) and received a terminal clean with ultraviolet (UV) light. Hospital beds remain in the room during cleaning and UV application. Cleaning practices are audited via environment-of-care rounds and direct observation. Mattress covers, used in all hospital beds, are semi-impermeable, and mattresses are not removed from the bed frames during cleaning and disinfection. At both hospitals, standard procedure involves inspecting mattresses annually, and all mattresses are rated for 5 years of use. Nearly all mattresses had been replaced in the 2 years prior to study initiation.

### Cohort description

We retrospectively identified all patients in tracked hospital beds from April 1, 2018, to August 31, 2019. We excluded beds for which we could not determine the location by the tracking system for >75% of eligible hospital nights. Additionally, we excluded the labor and delivery specialty beds because they rarely move to other units, and neonatal intensive care unit (ICU) beds, which were not tracked by the system. In total, 2.9% of beds were excluded from the analysis. From the electronic medical record, we extracted data on the patients’ hospital room and unit location throughout the admission, demographics, time at risk for *C. difficile* (defined as the number of hospital days preceding HO-CDI diagnosis or total length of stay for patients without the outcome), and receipt of antibiotics (other than vancomycin or metronidazole) or proton pump inhibitors (PPIs). We calculated the Elixhauser comorbidity score using *International Classification of Disease, Tenth Revision* (ICD-10) diagnostic codes.

### Exposure and outcome variables

**Figure 1.**
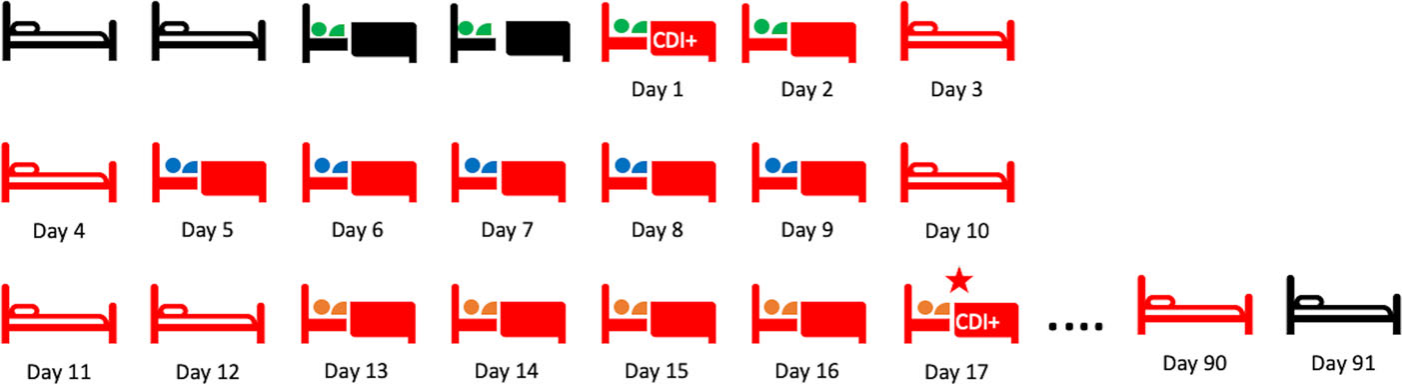
Exposure to a *Clostridioides difficile* “contaminated” bed. Beds are considered contaminated (red) starting with the positive *C. difficile* test of an occupant (green occupant). The bed remains contaminated for 90 days and “resets” every time a new patient with *C. difficile* is identified in that bed. Subsequent occupants (blue and orange) are considered to have an associated hospital-onset *C. difficile* infection (HO-CDI) if they develop their infection ≥3 days after being admitted and while residing in the contaminated bed or within 7 days of leaving the contaminated bed (orange occupant with star).

A patient was defined as being exposed to a potentially contaminated bed or room (referred to as “contaminated”) if, within the previous 7 days, they had resided in a hospital bed or a room that held a previous occupant with a positive *C. difficile* test in the previous 90 days (Fig. 1). The primary study outcome was HO-CDI, defined as a positive *C. difficile* test in a patient hospitalized for >3 days, in accordance with the National Health Safety Network (NHSN) definition. All *C. difficile* testing was performed using polymer chain reaction (PCR; Xpert *C. difficile* PCR assay, Cepheid, Sunnyvale, CA). To allow all beds to be tracked (and possibly exposed) using an initial 90-day run-in period, we only included patients as having the outcome of HO-CDI after July 1, 2018.

### Statistical analysis

We compared patients with and without the exposure using χ^2^ tests for categorical variables and the Wilcoxon-Mann-Whitney or Student *t* test for continuous variables, where appropriate. Using multivariable logistic regression, we evaluated the association between exposure to a contaminated bed and HO-CDI. Model covariates included time at risk for *C. difficile* and whether the patient required the ICU (assessed prior to HO-CDI or discharge, whichever occurred first). These covariates were chosen based on our a priori hypotheses and were statistically associated with both being in a contaminated bed (exposure) and HO-CDI (outcome). In a sensitivity analysis, we used the same multivariable model but changed the length of time we considered a bed to be contaminated after the prior occupant’s positive *C. difficile* test from 90 days to 60, 30, 14, and 7 days, respectively.

In a secondary analysis, we repeated the multivariable logistic regression using contaminated bed as our primary exposure and controlling for the same factors as above (ICU and time at risk). We also performed a counterfactual-based mediation analysis with a 4-way decomposition of the total causal effect of mediation and interaction to assess the contribution of a contaminated room to the relationship between a contaminated bed and HO-CDI.^16^ Thus, we divided the total causal effect of contaminated room on HO-CDI into 4 components: (1) a controlled direct effect not due to mediation or interaction, (2) a reference effect due to interaction alone, (3) the part of the total effect due to both interaction and mediation (ie, mediated interaction effect), and (4) the part of the total effect due entirely to mediation alone (ie, pure indirect effect).^16^ A mediator is an intermediate variable between the exposure and outcome that directly affects their relationship. Interaction occurs when the combined effect of an exposure and a second variable causes an increased effect on the outcome beyond the effect of either variable individually. Both mediation and interaction can occur simultaneously. All *P* values <.05 were considered statistically significant. The analysis was performed using SAS version 9.4 software (SAS Institute, Cary, NC).

### Ethical approval

The Emory University Institutional Review Board approved this study with a waiver of the patients’ informed consent.

## Results

We analyzed 25,032 hospital encounters representing 18,860 unique patients. Approximately half the encounters were with female patients (n = 12,938, 51.7%) and Black patients (n = 13,231, 52.9%). The median age was 61 years (IQR, 47–71) (Table 1). In total, 3,155 stool samples were collected and processed for *C. difficile*, of which 241 (7.6%) were positive. There were 237 (0.9%) hospital encounters with HO-CDI. A higher Elixhauser comorbidity score, longer time at risk, admission to the ICU or hospital B, and receipt of antibiotics or PPIs were all associated with being exposed to a contaminated bed (Table 1).

**Table 1.** Patient Characteristics Comparing Those Exposed and Not Exposed to a Contaminated Bed

Variable	Total Encounters (n=25,032), No. (%)^a^	Exposed to Contaminated Bed (n=6,287), No. (%)^a^	Not Exposed to Contaminated Bed (n=18,745), No. (%)^a^	*P* Value^b^
Age, median y (IQR)	61 (47–71)	61 (47–71)	61 (47–71)	.18
Sex, female	12,938 (51.7)	3,192 (50.1)	9,747 (52.0)	.09
**Race**				.18
Black	13,231 (52.9)	3,383 (53.8)	9,848 (52.5)	
White	10,373 (41.4)	2,543 (40.4)	7,830 (41.8)	
Other^c^	1,428 (5.7)	361 (5.7)	1067 (5.7)	
**Hospital**				<.0001
A	13,836	2467 (39.2)	8729 (46.6)	
B	11,196	3820 (60.8)	10016 (53.4)	
Time at risk, median d (IQR)^d^	5 (4-9)	6 (4-11)	5 (4-9)	<.0001
Required ICU during admission^d^	6,998 (28.0)	2,579 (41.0)	4,419 (23.6)	<.0001
Elixhauser score, median (IQR)	4 (2–5)	4 (3–6)	4 (2–5)	<.0001
Received antibiotics^d,e^	16,596 (66.3)	4,315 (68.6)	12,281 (65.5)	<.0001
Received proton pump inhibitor^d^	11,199 (44.7)	3,140 (49.9)	8,059 (43)	<.0001

Note. IQR, interquartile range; ICU, intensive care unit.

a Data are no. (%) unless otherwise specified.

b *P* value calculated using χ^2^ tests for categorical variables and the Wilcoxon-Mann-Whitney or Student *t* test for continuous variables.

c Includes American Indian or Alaskan Native, Asian, Native Hawaiian or other Pacific Islander, and unknown race.

d Prior to diagnosis of HO-CDI or discharge, whichever occurred first.

e Excluded both intravenous and oral metronidazole and vancomycin.

Patients who resided in a contaminated bed were more likely to have HO-CDI in the unadjusted analysis (odds ratio [OR], 1.8; 95% confidence interval [CI], 1.4–2.3) (Table 2). The median time between the prior occupant’s positive *C. difficile* test and the subsequent occupant’s HO-CDI diagnosis was 38.5 days (interquartile range [IQR], 22–68.5). The median time between the prior positive occupant’s last day in a contaminated bed and the subsequent occupant’s HO-CDI diagnosis was 34.5 days (IQR, 16.5–64.5). Beds moved among an average of 6.7 rooms during the study period.

**Table 2.** Odds of Hospital-Onset *C. difficile* Infection (HO-CDI) Following Exposure to a Contaminated Bed

Variable	Unadjusted OR (95% CI)	Adjusted OR^a^ (95% CI)
Exposure to contaminated bed^b^	1.8 (1.4–2.3)	1.5 (1.2–2.0)

Note. OR, odds ratio; CI, confidence interval; ICU, intensive care unit.

a Adjusted for ICU admission and time at risk prior to HO-CDI or discharge, whichever occurred first.

b Defined as having an occupant in the previous 90 days with a positive *C. difficile* test.

In a multivariable analysis, being exposed to a contaminated bed was significantly associated with HO-CDI (adjusted OR, 1.5; 95% CI, 1.2–2.0) after controlling for time at risk and ICU admission prior to HO-CDI or discharge (Table 2). In a sensitivity analysis using the same multivariable model, we adjusted the time we assumed the bed would remain contaminated for after a positive *C. difficile* test from 90 days to either 60, 30, 14, or 7 days, respectively. For all periods, exposure to a contaminated bed remained a predictor of HO-CDI (Table 3).

**Table 3.** Sensitivity Analyses Assessing Odds of Hospital-Onset *C. difficile* Infection (HO-CDI) While Varying the Length of Time We Considered the Bed to be Contaminated for After Prior Occupant Tested Positive for *C. difficile*

Variable	60 Days Adjusted OR^a^ (95%CI)	30 Days Adjusted OR^a^ (95% CI)	14 Days Adjusted OR^a^ (95% CI)	7 Days Adjusted OR^a^ (95% CI)
Exposure to contaminated bed	1.3 (1.0–1.8)	1.6 (1.1–2.2)	1.6 (1.0–2.4)	1.5 (0.9–2.7)

Note. OR, odds ratio; CI, confidence interval; ICU, intensive care unit.

a Adjusted for ICU admission and time at risk prior to HO-CDI or discharge, whichever occurred first.

Most patients (68.4%) who were exposed to a contaminated hospital bed were also exposed to a contaminated hospital room. Exposure to a contaminated room was associated with HO-CDI in both an unadjusted analysis (OR, 1.9; 95% CI, 1.5–2.5) and an adjusted analysis, controlling for the same variables used in the bed model (OR, 1.5; 95% CI, 1.1–1.9). In a secondary analysis, 62% (95% CI, 24%–100%) of the relationship between HO-CDI and exposure to a contaminated bed was due to both mediation and interaction between the room and bed status (Fig. 1 and Supplementary Tables online).

## Discussion

In this study of over 25,000 hospital encounters, we demonstrated that residing in a hospital bed that previously had an occupant with *C. difficile* remained a risk factor for HO-CDI in multivariable analysis. This association persisted even after varying the amount of time we assumed the bed would remain contaminated following the prior occupant’s *C. difficile* diagnosis. Residing in a hospital room that previously had an occupant with *C. difficile* is also a risk factor for HO-CDI, and the association between contaminated bed and HO-CDI is in part explained by the fact that most patients in a contaminated bed were also exposed to a contaminated room.

These results are consistent with previous research examining the relationship between a prior room occupant with a multidrug-resistant organism (MDRO) and risk of infection or colonization with a MDRO for the subsequent room occupant.^17^ In 2010, Shaughnessy et al^13^ examined the association between hospital room and CDI and found that those with CDI were more likely to have been in a room where the prior occupant had CDI (hazard ratio 2.35, 95% CI 1.21–4.54). This study was limited to critically ill patients at a single hospital, and their analysis only accounted for whether the immediate prior room occupant had CDI. The analysis did not specify whether the same bed was used between patients. Sood et al^14^ recently described similar increased odds of CDI (OR, 1.27; 95% CI, 1.12–1.44) across multiple hospitals when a patient was exposed to a contaminated room, with an increasing risk of CDI with each day exposed to the contaminated room. Lastly, Freedberg et al^9^ found that patients are at risk for CDI when the prior bed occupant received antibiotics. Our study builds on these prior investigations and, to our knowledge, is the first to examine the risk of HO-CDI associated with being in a hospital bed that previously had an occupant with *C. difficile*.

The odds of developing HO-CDI with exposure to a contaminated bed minimally increased in sensitivity analyses in which we decreased the amount of time a bed was considered contaminated, thus decreasing the time between prior occupant’s positive *C. difficile* test and the subsequent occupant’s diagnosis of HO-CDI. Estimates for the smaller exposure windows were less precise, however, due to the decreasing number of patients considered exposed to a contaminated bed, although the effect estimate (adjusted OR, 1.5) remained consistent. Overall, the sustained the association between contaminated bed and HO-CDI speaks to the robustness of *C. difficile* spores and their ability to survive on environmental surfaces for an extended period. This research has important implications for understanding transmission dynamics of *C. difficile* throughout a hospital and indicates the need for improved cleaning and disinfection protocols of the hospital bed and the surrounding healthcare environment. Real-time tracking of hospital beds also has the potential to aid in outbreak investigations of HO-CDI by identifying connections (eg, shared beds) between patients that traditional epidemiologic methods may miss.

Our study had several limitations. Certain beds may reside in the same units consistently, making those beds more likely to have HO-CDI occupants primarily due to the types of patients occupying those units (eg, hematopoietic stem-cell transplant floors or ICUs). We limited our analysis to beds for which we could identify a tracked location in 75% of the eligible nights. However, we do not believe the excluded beds significantly differ from those included. *C. difficile* testing during the study period was performed using PCR, which may have led to an overdiagnosis of HO-CDI but is likely a more sensitive marker of potentially contaminated hospital beds. We also did not account for clinical severity, which could affect the degree of spore transmission to the next bed occupant. Similarly, our outcome of HO-CDI did not capture asymptomatic acquisition of *C. difficile* or episodes of *C. difficile* that occurred after hospital discharge. However, if hospital bed or room does increase odds of *C. difficile* acquisition, this limitation should bias our results toward the null. The relationships among the hospital bed, hospital room, and HO-CDI are complex. We assumed that being in a contaminated room is on the causal pathway between contaminated bed and HO-CDI; however, it is possible that the contaminated room is a confounder in this relationship. A contaminated bed may also mediate the relationship between contaminated room and HO-CDI, but our mediation analysis remains valid because we also allowed for interaction. Although our analysis included only single-bedded rooms, we believe that our results are applicable to facilities with semiprivate rooms and that having more beds in a room would increase the risk for all occupants of a contaminated room. Lastly, other factors likely exist that we did not control for, including additional components of the healthcare environment and prevalence of *C. difficile* in the community.

National, regional, and institutional efforts have aimed to reduce HO-CDI through minimizing inappropriate antibiotic prescribing and improving infection prevention practices. Our findings suggest that there may be transmission of *C. difficile* from the hospital bed to a patient, even up to 90 days after the original patient was diagnosed with *C. difficile*. Further studies involving genomic sequencing could more directly link possible bed contamination and developing HO-CDI. Human-factor analyses could also elaborate on the interactions that healthcare personnel have with the hospital room, bed, and other surfaces to determine which parts of the healthcare environment contribute the most to transmission of HO-CDI. New technologies or cleaning and disinfection methods that can better eradicate *C. difficile* spores from a hospital bed and/or the surrounding healthcare environment may lead to significant reductions in healthcare transmission of *C. difficile* and decrease rates of HO-CDI.

## Supplementary material

To view supplementary material for this article, please visit https://doi.org/10.1017/ice.2023.254
